# The ground beetle tribe Platynini Bonelli, 1810 (Coleoptera, Carabidae) in the southern Levant: dichotomous and interactive identification tools, ecological traits, and distribution

**DOI:** 10.3897/zookeys.1044.62615

**Published:** 2021-06-16

**Authors:** Thorsten Assmann, Esteve Boutaud, Jörn Buse, Claudia Drees, Ariel-Leib-Leonid Friedman, Ingmar Harry, Fares Khoury, Eylon Orbach, Ittai Renan, Constantin Schmidt, Kilian Schmidt, David W. Wrase, Pascale Zumstein

**Affiliations:** 1 Institute of Ecology, Leuphana University Lüneburg, Universitätsallee 1, D-21335 Lüneburg, Germany Leuphana University L&uuml;neburg Germany; 2 Ecosystem Monitoring, Research and Wildlife Conservation (SB 23 Invertebrates and Biodiversity), Black Forest National Park, Kniebisstraße 67, D-72250 Freudenstadt, Germany Black Forest National Park Freudenstadt Germany; 3 School of Life Sciences, University of Sussex, Brighton, BN1 9QG, United Kingdom University of Sussex Brighton United Kingdom; 4 Steinhardt Museum of Natural History, Tel Aviv University, Klauzner 12, Tel Aviv, IL-69978, Israel Tel Aviv University Tel Aviv Israel; 5 Office for Conservation Biology ABL, Egonstraße 55, D-79106 Freiburg, Germany Office for Conservation Biology Freiburg Germany; 6 Department of Biology and Biotechnology, American University of Madaba, P.O.Box 2882, Amman, JO-11821, Jordan American University of Madaba Madaba Jordan; 7 Remez St. 49, IL-36044 Qiryat Tiv’on, Israel Unaffiliated Qiryat Tiv'on Israel; 8 Gartenstr. 8, 21354 Bleckede, Germany Unaffiliated Bleckede Germany; 9 Oderstraße 2, D-15306 Gusow-Platkow, Germany Unaffiliated Gusow-Platkow Germany

**Keywords:** *Agonum*, *Atranus*, *Anchomenus*, ecological traits, interactive key, *Olisthopus*, *Orthotrichus*, power of dispersal, phenology, winter ponds, Xper3

## Abstract

The carabids of the tribe Platynini from the southern Levant (Egypt: Sinai Peninsula, Israel, Jordan) and adjacent regions of Egypt, Lebanon, Syria, Iraq, and Saudi Arabia are reviewed in terms of species taxonomy, ecological, distributional traits, and conservation biology. In addition to a classical dichotomous identification key to the 14 species of the region, identification tools are made freely available via the Xper3 knowledge database “Platynini, southern Levant”. Besides an interactive identification key, a matrix with character states for the species and single access identification keys are available. A database including all available records from the southern Levant is also provided. First faunistic records are recorded for *Anchomenusdorsalisinfuscatus* from Sinai (Egypt), *Olisthopusfuscatus* from Lebanon and Iraq, and for *O.glabricollis* from Iraq. Threatened species are discussed, also with regard to the reasons of their decline. The majority of species lives in wetlands, especially on the shore of winter ponds and streams, which have been extremely degraded in the last decades.

## Introduction

The Platynini, a tribe of ground beetles, are widespread in the southern Levant. The taxonomy of some species and subspecies, including new descriptions and synonyms, has been clarified just in the last two decades (Schmidt and Liebherr 2009; Schmidt 2014). From an ecological and conservation biological point of view, this tribe is important, as most taxa prefer habitats with high ground water tables. Such habitats are in sharp decline in the southern Levant due to human impacts. Expansion of structures and intensified land-use over the last few decades led to an effective loss of land that could serve as habitat for plant and animal species (Bittner and Sofer 2013). For many Platynini species with a preference for riparian habitats, this habitat loss was achieved by drainages, channeling of streams, and also by water withdrawal from streams for drinking water and agricultural uses. Because the Platynini include taxa that are exclusive to this region, a responsibility arises for the worldwide conservation of the given taxa.

In order to support ecologists, environmentalists, and conservationists, but also citizen scientists, we have developed identification keys for this tribe. We present these taxonomic tools in two forms: a “classical” dichotomous key and an interactive key with further identification tools like character matrices.

In the pioneer phase of entomological exploration of the southern Levant, some species of Platynini were already recorded (e.g., Bodenheimer 1932; Schatzmayr 1936; Bodenheimer 1937). Some of them were not verified in the collections, and subsequent works lack information on these (e.g., Schmidt 2017). We have analyzed the collections available to us from this region, compiled ecological and distributional traits. This information enabled us to provide preliminary information on threats to the Platynini species from the southern Levant.

## Materials and methods

This study is based on the examination of specimens (i) observed and/or collected during field trips of the authors in Israel, Jordan and Egypt (between 2004 and 2020), (ii) collected in context of ecological surveys (e.g., Timm et al. 2009), and/or (iii) stored in entomological collections (incl. historical collections). In addition, records of species have been critically compiled from available literature (e.g., Bodenheimer 1932; Schatzmayr 1936; Bodenheimer 1937; El-Moursy et al. 2001; Abdel-Dayem 2004; Chikatunov et al. 2004; Chikatunov et al. 2006; Schmidt and Liebherr 2009; Wrase 2009; Schmidt 2014; Nasir and Katbeh-Bader 2017). We studied more than 800 Platynini specimens from the southern Levant, but material from Lebanon and Syria was very limited.

Abbreviations of collections (used also in Suppl. material 1):

**CAB** Working collection Assmann, Asendorf (formerly Bleckede) (part of ZSM, Germany);

**CBS** Working collection Buse, Seebach, Germany;

**CEL** Working collection Boutaud, Lüneburg, Germany;

**CIH** Working collection Harry, Freiburg;

**COQ** Working collection Orbach, Qiryat Tiv’on, (will be transferred to SMNHTAU, Israel);

**CWGP** Working collection Wrase, Gusow-Platkow (part of ZSM, Germany);

**SMNHTAU**Steinhardt Museum of Natural History, Tel Aviv University, Tel Aviv, Israel;

**ZSM**Zoological State Collection Munich (Zoologische Staatssammlung München), München, Germany.

The basic approach to identify ecological and distributional traits and threats follows our previous work (Assmann et al. 2018). Numbers in parentheses after hindwing classification refer to the number of dissected specimens (cf. Assmann et al. 2012). Some ground beetle species occur in ‘batha’ (*φρύγανα*, phrygana in Greece and Turkey), a habitat type characterized by Mediterranean semi-shrubs (e.g., *Sarcopotheriumspinosum*) after abandonment and some grazing pressure (Danin 1988; Albert et al. 2004).

We prepared the photos with the microscope and camera mentioned in the previously cited publications (Assmann et al. 2018) or with a Lumix GX 80 and a Leica Macro-Elmarit 45 mm. Stacking (up to ca. 100 layers) was done with the software Picolay (www.picolay.de).

### Interactive identification tools

We created an interactive key in the Xper3 version 1.5.2, a collaborative web-based platform (https://www.xper3.fr) (Pinel et al. 2017; Saucède et al. 2020). The manual of Xper3 is somewhat cryptic, partly in French. Better introductions about the workflow and the applicability are given by Jouveau et al. (2018) and Klimmek and Baur (2018).

As “items”, the species are incorporated into the database (“Platynini, southern Levant”). The characters (“descriptors”) and their states have been adopted from the dichotomous identification key. These shape the descriptive model of our Xper3 knowledge base. If character states are not applicable, they are indicated as “Unknown values”. The pictures of the dichotomous identification key are also hosted as *.png files on the homepage zenodo.org. We checked completeness of the database using the inbuilt tools of Xper3. Two taxonomic laypersons used several versions of the interactive key and supported decisions on how to weigh the characters. We modified the single access key by suggestions of the laypersons regarding ‘weight use’ for the characters, and the prioritization of characters with fewer states.

## Results

### Recorded species

Altogether, we could verify more than 800 specimens of platynines from the southern Levant. We can present here first faunistic records for countries of the southern Levant: *Anchomenusdorsalisinfuscatus* from Sinai (Egypt), *Olisthopusfuscatus* from Lebanon and Iraq, and *O.glabricollis* from Iraq. If geographic coordinates are not given on the labels, we have determined them according to the name of the localities. These data may not be as accurate as the measured ones and are marked in yellow (Suppl. material 1).

Following the database and our literature survey, a total of 13 species occurs in the southern Levant, and one additional species may occur in Northwest Syria. In the following key, we list the latter species in parentheses. We incorporated the 14 species in both identification keys, the classic dichotomous identification key and the interactive key.

### Characterization of Platynini in the southern Levant

**Figures 1–4.**
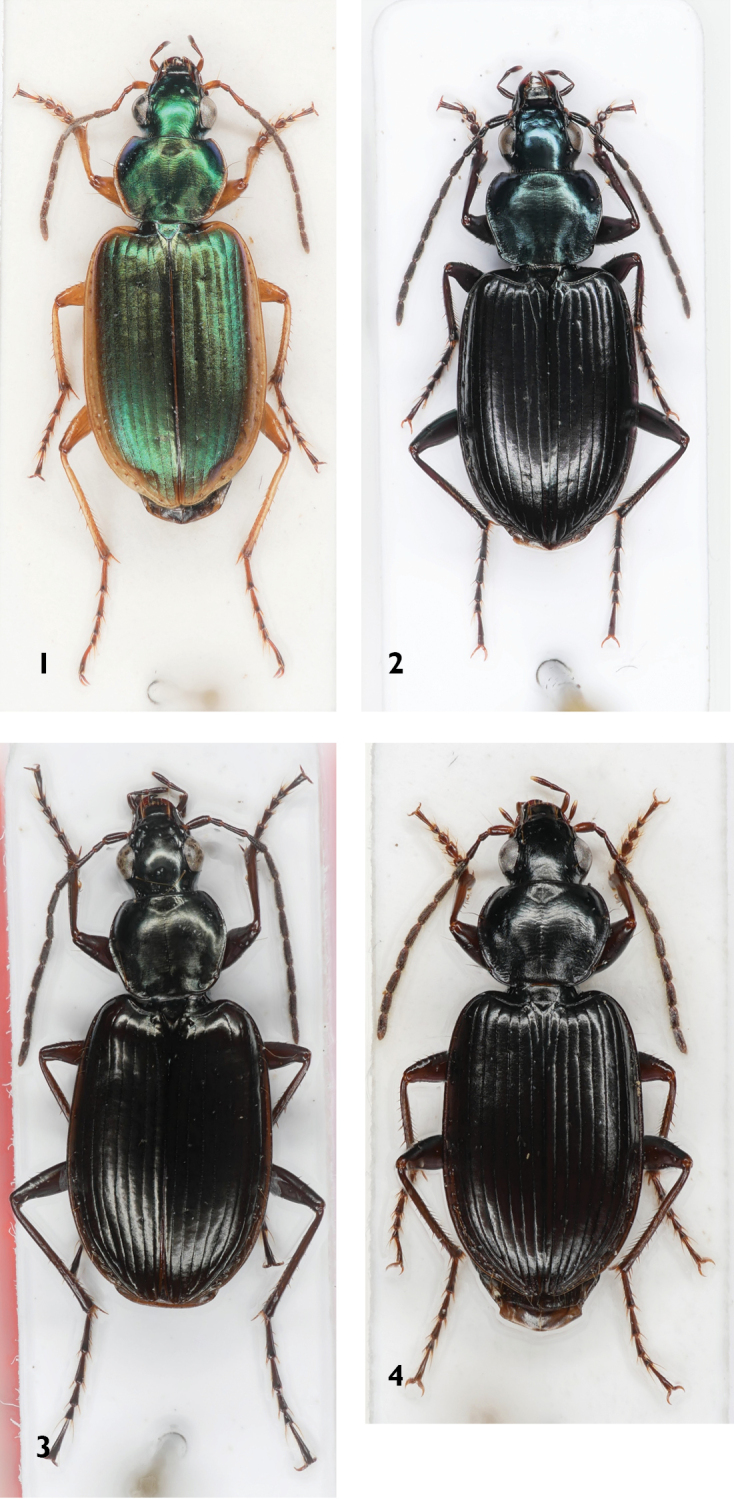
Platynini species **1***Agonummarginatum***2***Agonummesostictum***3***Agonummonachumsyriacum***4***Agonumnigrum*.

**Figures 5–8.**
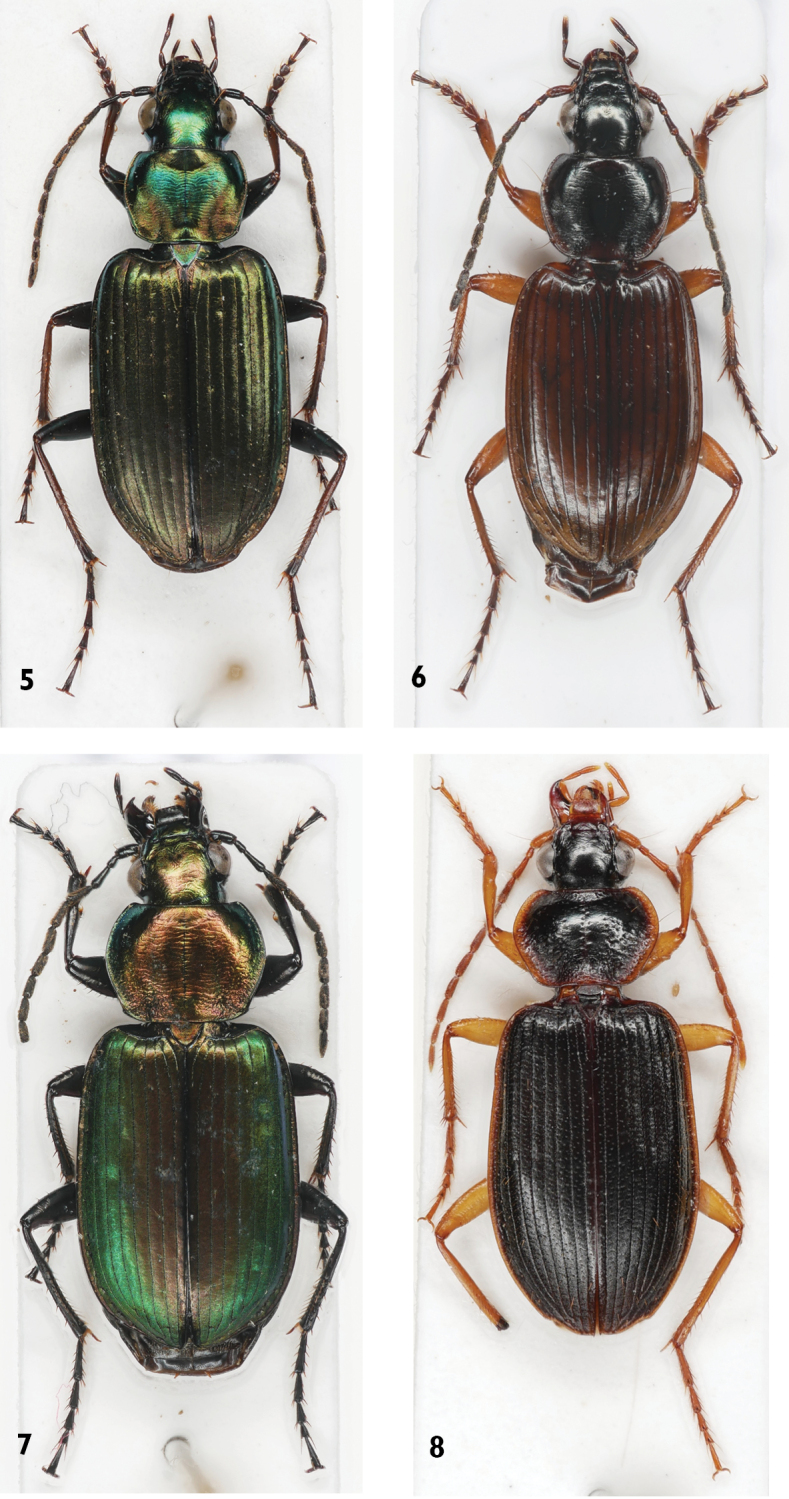
Platynini species **5***Agonumrugicolle***6***Agonumsordidum***7***Agonumviridicupreum***8***Orthotrichuscymindoides*.

**Figures 9–12.**
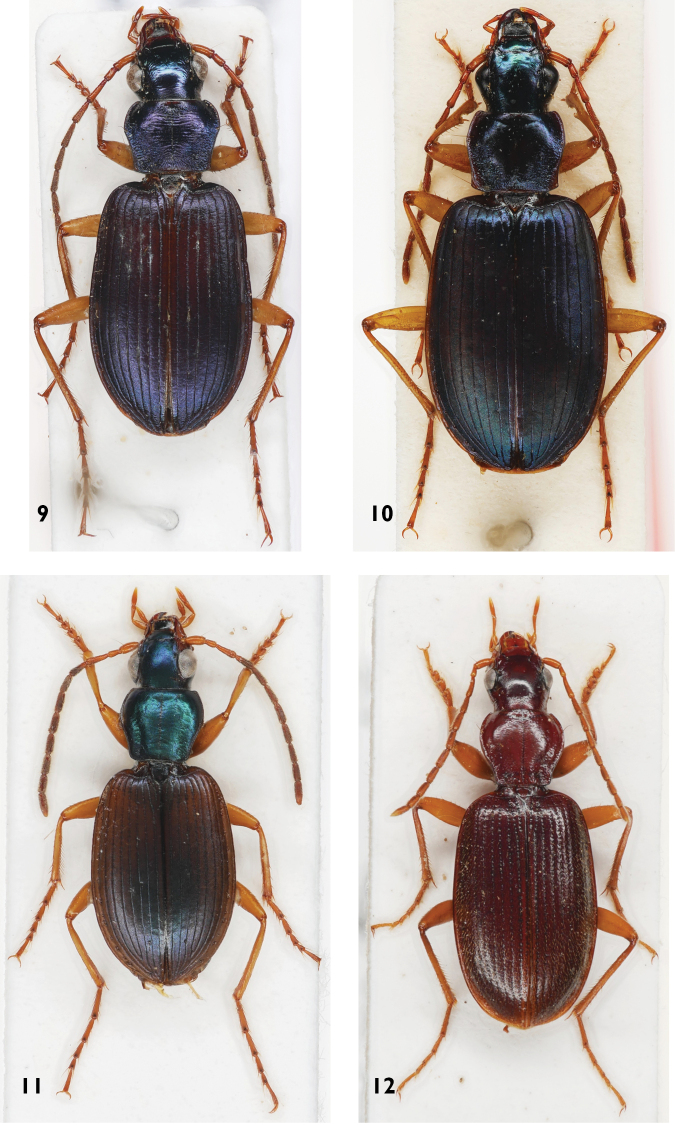
Platynini species **9***Anchomenusalcedo***10***Anchomenusbellus***11***Anchomenusdorsalisinfuscatus***12***Atranusruficollis*.

**Figures 13, 14.**
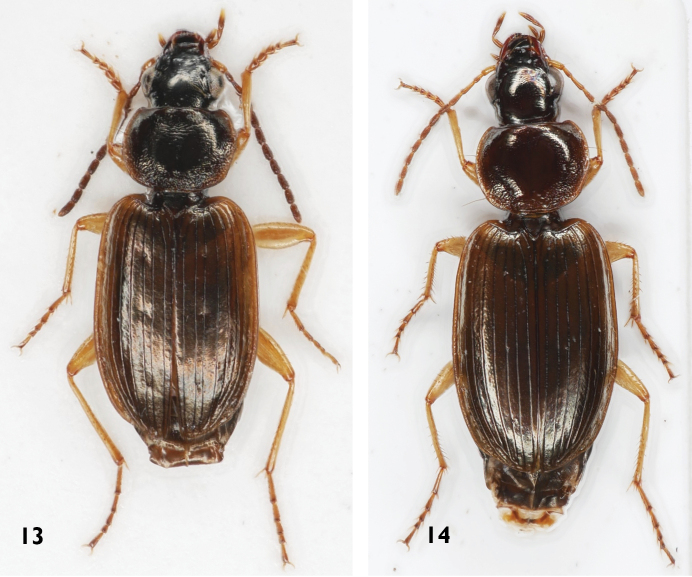
Platynini species **13***Olisthopusfuscatus***14***Olisthopusglabricollis*.

Within the Harpalinae, Platynini can be characterized as follows:

Medium sized species of slender and flat habitus, often with noticeable metallic luster. Head with two pairs of supraorbital setae. Mandible scrobe (groove on the basal half of the mandible laterally) without a seta. Antennae pubescent from the 2^nd^, 3^rd^, or 4^th^ antennomeres. Penultimate labial palpomere with two setae on its anterior margin. Process of prosternum not margined apically. Protibia apically with deep emargination (antenna cleaner) not enlarged. Tarsi on the upper side rarely pubescent. Ventral edges of claws smooth. Both parameres of the median lobe of aedeagus spatulate and ovate. In addition, Figs 1–14 help to identify representatives of the tribe in the southern Levant.

### Dichotomous identification key

**Table:** 

1	Upper side with numerous hairs, especially on pronotum and elytra	**2**
–	Upper side without hairs, only regular setae (supraorbital setae, series umbilicata, etc.)	**3**
2	Pronotum cordiform, almost as long as wide (Fig. 21). Hind angles of pronotum obtuse, somewhat upwards bent. Apical margin of elytra evenly rounded (Fig. 22). Reddish to dark brown, head sometimes darker and pronotum brightened. (Fig. 12). 5.5–8.0 mm	***Atranusruficollis* (Gautier des Cottes, 1858)**
–	Pronotum clearly wider than long (Fig. 20). Hind angles completely rounded. Apical margin of elytra sinuous, with small tooth at end of suture (Fig. 23). Body dark brown, antennae and legs and margins of pronotum and elytra reddish brown. (Fig. 8). 9–12 mm	***Orthotrichuscymindoides* (Dejean, 1831)**
3	Labrum clearly convex; apical margin rounded; mandibles shorter, ca. 2× as long as labrum (Fig. 27). Mentum without tooth (see https://doi.org/10.5281/zenodo.4430705)	**4**
–	Labrum flat or slightly convex; apical margin almost straight (sometimes slightly convex or concave); mandibles longer, in most species > 2.5 × as long as labrum (Figs 28–30). Mentum with tooth (see https://doi.org/10.5281/zenodo.4430699)	**5**
4	Pronotum wider, ca. 1.3× wider than long; lateral bead broader, punctuation stronger (Fig. 18). At least part of first 3 antennomeres dark. Setiferous punctures of 3^rd^ elytral stria large and adjacent intervals clearly depressed. With or without pronounced depression of 5^th^ stria and adjacent intervals in apical third of elytra (Fig. 13). 5.0–6.5 mm	***Olisthopusfuscatus* Dejean, 1828**
–	Pronotum longer, ca. 1.2× wider than long; lateral bead slender, punctuation weaker (Fig. 19). At least first 3 antennomeres yellowish brown. Setiferous punctures of 3^rd^ elytral stria small, and adjacent intervals not extraordinarily depressed. Without depression of 5^th^ stria and adjacent intervals in apical third of elytra (Fig. 14). 5–6.5 mm	***Olisthopusglabricollis* (Germar, 1817)**
5	Hind angles of pronotum ca. rectangular, sometimes produced in sharp denticle, sometimes bases of pronotum somewhat convex. Upper side colorful either with blue to violet metallic luster or head and pronotum with green or blue metallic luster and elytra matt brown with darkening in central and apical part (Figs 9–11, 21)	**6**
–	Hind angles of pronotum entirely rounded or obtuse, sometimes slightly produced into minute denticle (Figs 1–8, 15–17)	**8**
6	Head and pronotum with green or bluish green metallic luster, elytra matt brown with darkening in central and apical part. Hind angles of pronotum without seta (Fig. 11). 5.0–7.0 mm	***Anchomenusdorsalisinfuscatus* Chevrolat, 1854**
–	Head, pronotum and elytra with blue to violet luster (Figs 9, 10). Hind angles of pronotum with seta. Longer than 8.5 mm	**7**
7	First antennomere in apical part enlarged, behind a small constriction (“trumpet-shaped”). Pronotum 1.26–1.38 wider than long (Fig. 9). 9.0–10.5 mm	***Anchomenusalcedo* Schmidt, 2014**
–	First antennomere regularly shaped, in apical part neither with a constriction nor widened at apex. Pronotum 1.16–1.27 wider than long (Fig. 10). 8.7–10.3 mm	**(*Anchomenusbellus* Schmidt, 2014)**
8	Upper side green and/or coppery; with broad yellow elytral margin; yellow coloration on pronotal margin narrower (Fig. 1). 8.5–10.5 mm	***Agonummarginatum* (Linnaeus, 1758)**
–	Upper side variable, from black and brown to vivid metallic luster, but without yellow margin on elytra or pronotum (Figs 2–7)	**9**
9	Third elytral intervals with (5-) 6 (-7) setiferous punctures. Upper side with metallic luster, mostly elytra green and forebody (head, pronotum) reddish or coppery, rarely black (Fig. 7). 8–10 mm	***Agonumviridicupreum* (Goeze, 1777)**
–	Third elytral intervals with (2-) 3 (-4) setiferous punctures. Upper side of different color	**10**
10	Elytra brownish, femora reddish to pale brown; tibiae darker. Apex of 3^rd^ antennal segment with additional (sometimes only few) small hairs beside regular erect setae (Fig. 6). 7–9.5 mm	***Agonumsordidum* Dejean, 1828**
–	Elytra with metallic luster or black or dark brown to black. Apex of 3^rd^ antennal segment only with regular setae	**11**
11	Larger species (> 8.5 mm) with small and wide pronotum (mostly more than 1.3× wider than long). Microsculpture of elytra isodiametric and strongly developed, also in males. Vivid metallic green or bronze, rarely black. With depression in 5^th^ stria and adjacent intervals of apical third of elytra (Fig. 5). 8.5–11.5 mm	***Agonumrugicolle* Chaudoir, 1846**
–	Smaller species up to 9.0 mm with larger and slender pronotum (mostly < 1.3 × wider than long). Microsculture of elytra isodiametric, but less developed. Never vividly metallic colored, elytra dark brown to black, completely without or with slight metallic luster	12
12	Pronotum wider, 1.2 to 1.3× wider than long, lateral margin continuously rounded between maximum width and hind angles (Fig. 17). Coloration dark brown to black, first antennal segments and tibiae, sometimes femora brown. Without any metallic luster. Without or with slight depression of 5^th^ stria and adjacent intervals in apical third of elytra (Fig. 4). 7–8.5 mm	***Agonumnigrum* Dejean, 1828**
–	Pronotum narrower, < 1.2× wider than long, lateral margin from maximum width towards hind angles slightly straight or slightly concave (Figs 15, 16). Coloration similar, but legs and antennae homogeneously paler or darker brown. Without or only a slight metallic hue on whole upper side or forebody (head, pronotum). Without or with a clearly visible depression of 5^th^ stria and adjacent intervals in apical third of elytra (Figs 24, 25)	**13**
13	Pronotum 1.1 to 1.2× wider than long, lateral margin from maximum width towards hind angles straight or very slightly concave, hind angles less rounded, fore angles pronounced (Fig. 15). Without depression of 5^th^ stria and adjacent intervals in apical third of elytra (Fig. 25). Legs and antennae shorter. Almost dark brown to black, tibiae sometimes paler, without or with faint metallic luster (Fig. 2). 7.5–9.0 mm	***Agonummesostictum* (Bates, 1889)**
–	Pronotum ≤ 1.1× wider than long, lateral margin from maximum width towards hind angles straight, hind angles more rounded, fore angles stronger rounded (Fig. 16). Clearly visible depression of 5^th^ stria and adjacent intervals in apical third of elytra (Fig. 24). Legs and antennae longer. Elytra, first 3 antennal segments and legs brown, upper side with faint green metallic luster (Fig. 3). 8.5–9 mm	***Agonummonachumsyriacum* Schmidt & Liebherr, 2009**

The dichotomous identification key has 13 alternatives. The number of alternatives or clusters of alternatives, respectively, that have to be considered for the identification of a single species ranges from two to nine.

**Figures 15–17.**
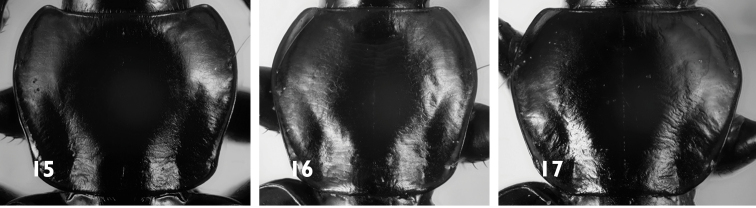
Pronotum of *Agonum* species **15***A.mesostictum***16***A.monachumsyriacum***17***A.nigrum*.

### Interactive, web-based identification tools

The interactive key is based on 16 characters with the number of character states ranging from 2 to 12 (Table 1). All, but two characters are categorical. The numerical characters are number of setiferous punctures in the 3^rd^ elytral interval and body length.

**Figures 18–21.**
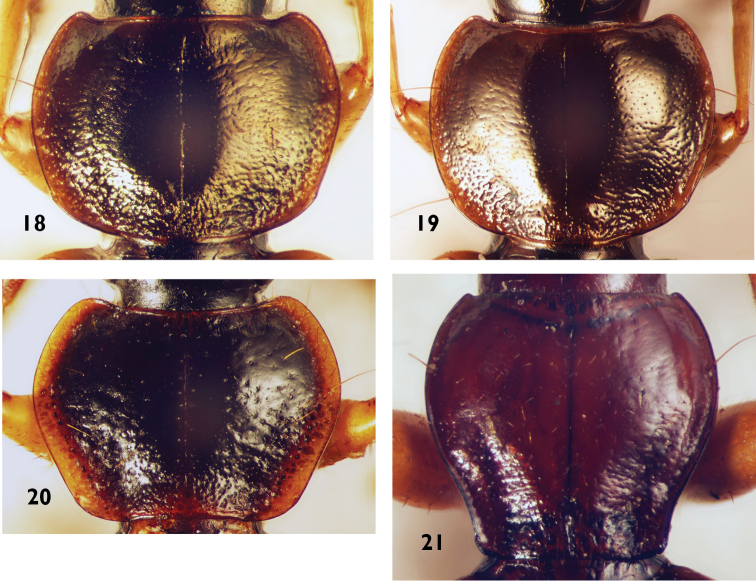
Pronotum of Platynini species **18***Olisthopusfuscatus***19***Olisthopusglabricollis***20***Orthotrichuscymindoides***21***Atranusruficollis*.

The states for the characters punctuation of pronotum and the number of setiferous punctures in the 3^rd^ elytral interval are not available for all species. In these cases, additional photographs would have been necessary to make the hard-to-recognize character states of these species clear to the users of the key. The number of setiferous punctures in the 3^rd^ elytra interval is difficult to detect in strongly pubescent species; and the intensity of the punctation of the pronotum can be inferred by a user only by specimen comparisons or with Figs 18, 19.

**Figures 22, 23.**
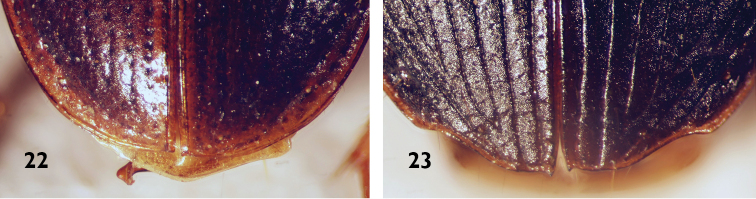
Apical margins of elytra of Platynini species **22***Atranusruficollis***23***Orthotrichuscymindoides*.

**Table 1.** Overview of the characters, number of their states, and number of species for which the character states are applicable in the Xper3 database “Platynini, southern Levant”.

Character (“descriptor“)	Number of character states (categories)	Number of species for which character states are applicable
Body length (numerical)	Variable	14
Coloration of upper side (head, pronotum, elytra) (categorical)	13	14
Metallic luster of upper side (categorical)	3	14
Coloration of legs (categorical)	4	14
First antennomere (categorical)	2	14
Labrum (categorical)	2	14
Mandibles (categorical)	2	14
Pronotum hind angles (categorical)	2	14
Pronotum lateral margin (categorical)	3	14
Pronotum proportion (categorical)	4	14
Pronotum punctation (categorical)	2	3
Hairs on upper side (pronotum, elytra) (categorical)	2	14
3^rd^ elytral interval, number of setiferous punctures (numerical)	Variable	12
5^th^ elytral stria and adjacent intervals (categorical)	2	14
Apical margin of elytra (categorical)	2	14

Without the option for applying these character states in some species, the interactive key helps to identify the species more quickly than the dichotomous one: If the character with the most states (esp. coloration of upper side) is included for the first decision, only 1 to 3 decisions are necessary for the identification of a species. However, if characters with a low number of states are prioritized, the number of decisions increases but remain lower than for the dichotomous identification key and ranges from 2 to 5.

Our Xper3 knowledge data base “Platynini, southern Levant” offers the option for generating single access keys as an alternative to the interactive key. Many of these keys are shorter than our dichotomous key, but it comes at the cost of having the same species listed two or three times within these keys. This results from the variability of coloration, but also other morphological characters, e.g., the deepened or not deepened 5^th^ interval at the apex of the elytra. Moreover, it bases on the coloration of the upper side and seems not to be the most appropriate version of an identification using the character state matrix of the database ‘Platynini, southern Levant’.

After a discussion with the two laypersons who tested several keys generated by Xper3, we weighted four characters strongly, two weakly, and all others medium. By using the options ‘use weights’ for the characters and ‘prioritize characters with fewer states’ we generated a single access key which has 17 alternatives (Suppl. material 2). The number of decisions, which are necessary for an identification, ranges from 2 to 6 and is lower than the one of the dichotomous key.

The interactive identification key is available at https://www.xper3.fr/xper3GeneratedFiles/publish/identification/7440398067574196430/.

The database is available at https://www.xper3.fr/xper3GeneratedFiles/publish/html/9050622959361543452/

The sdd file (in xml format) with the information incorporated in the knowledge database ‘Platynini, southern Levant’ is available in Suppl. material 3.

**Figures 24–26.**
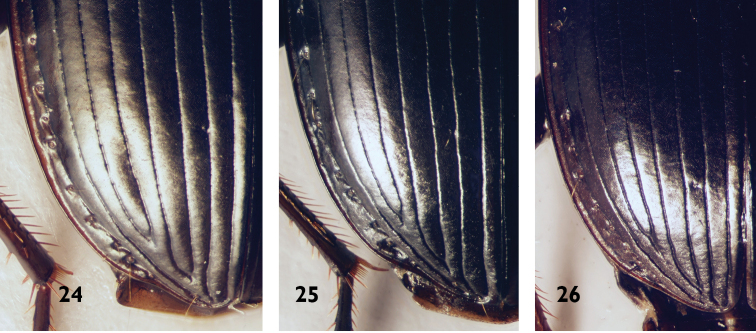
Apical part of left elytron with 5^th^ stria and adjacent intervals of Platynini species **24***Agonummonachumsyriacum*: depressed **25***A.mesostictum*: not depressed **26***A.nigrum*: not depressed, but see Fig. 4 for an *A.nigrum* individual with depression.

**Figures 27–30.**
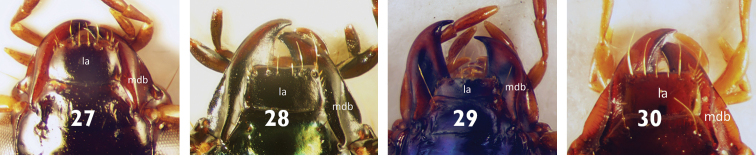
Apical part of Head of Platynini species **27***Olisthopusglabricollis***28***Agonumrugicolle***29***Anchomenusalcedo***30***Atranusruficollis*. Abbreviations: **la**: labrum, **mdb**: mandible.

### Species accounts

#### Agonum (s. str.) marginatum

Taxon classificationAnimaliaColeopteraCarabidae

(Linnaeus, 1758)

3EA8BF35-1840-55F2-88DD-0AFB99C0FA98

##### Dispersal power.

Fully winged and flight-active; able to quickly colonize restored habitats (Lindroth 1945; Trautner and Rietze 2017; pers. obs.).

##### Habitat.

**Figures 31–34.**
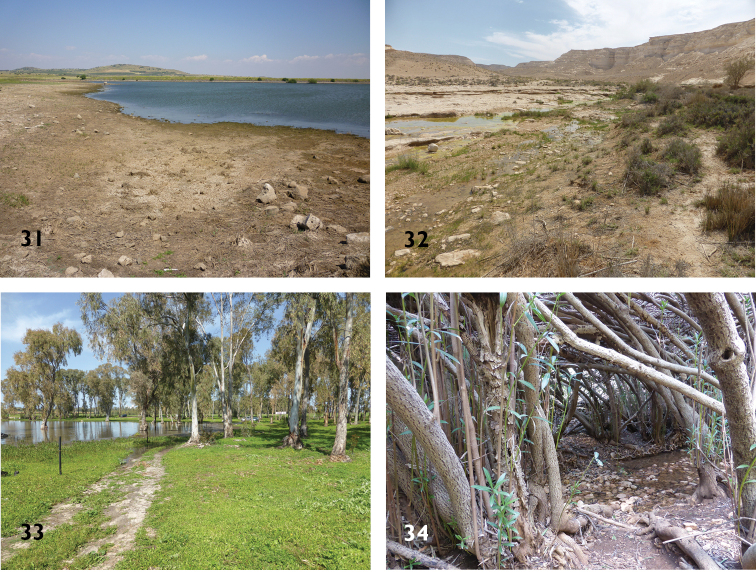
Habitats of Platynini species in the southern Levant **31** Merom Golan (Golan Heights): water reservoir, habitat of *Agonumrugicolle*, *A.sordidum*, *A.marginatum*, *A.viridicupreum*, *A.nigrum*, and *Anchomenusdorsalisinfuscatus***32** Ein Akev (Central Negev): headwater stream in desert, habitat of *Agonumnigrum***33** Breikhat Ya’ar (Coastal Plain, east of Hadera): winter pond, habitat of *Agonummonachumsyriacum*, *A.viridicupreum*, *A.nigrum*, and *Anchomenusdorsalisinfuscatus***34** Nahal Bezet (Upper Galilee): softwood floodplain woodland with Oleander (*Neriumoleander*), habitat of *Atranusruficollis*.

Stream banks and margins of winter ponds and artificial water reservoirs where vegetation is mostly sparse (Fig. 31).

##### Phenology.

Spring breeder (Lindroth 1945), adults hibernate (own observation).

##### Distribution range.

Throughout Europe (without North Scandinavia), southwards to North Africa, eastwards to Cyprus, Asia Minor, and southern Levant (Schmidt 2017).

##### Distribution in the southern Levant.

Already listed by Bodenheimer (1937) for the southern Levant without exact localities. Recently recorded only from the northern part of Israel (Upper Galilee, Golan Heights, Northern Coastal Plain); there are two old records from the Southern Coastal Plain (Miqwe Yisrael 1941) and Judean Hills (Hartuv 1925).

##### Conservation.

In most parts of the distribution range abundant, not threatened (e.g., Schmidt et al. 2016).

#### Agonum (s. str.) mesostictum

Taxon classificationAnimaliaColeopteraCarabidae

(Bates, 1889)

452C027D-5C02-5249-BAD0-709AD81DF6A4

##### Dispersal power.

Fully winged (n = 2). No flight observation known.

##### Habitat.

We know the habitat only from one site in Iran: partly overgrown gravel banks along streams at ca. 2350 m a.s.l. Also in Kyrgyzstan at high altitude (2000 m).

##### Phenology.

Unknown.

##### Distribution range.

From Georgia to Central Asia and Pakistan (Schmidt and Liebherr 2009).

##### Distribution in the southern Levant.

Schmidt and Liebherr (2009) indicate an old specimen, labelled “Syria”. We do not know any record from the southern Levant.

##### Taxonomic notes.

For their description of the species, Schmidt and Liebherr (2009) had mostly old material and mentioned that the beetles do not have a metallic luster. But, at least one male from recent years has a head and pronotum with a faint metallic luster (sheen) (Fig. 2).

#### Agonum (s. str.) monachum syriacum

Taxon classificationAnimaliaColeopteraCarabidae

Schmidt & Liebherr, 2009

03964FA4-A59E-5E6E-BEBC-D57B19835F74

##### Dispersal power.

Fully winged and flight-active (pers. obs.).

##### Habitat.

Known only from winter ponds (Fig. 31) and intact large wetlands (e.g., swamps north of the Sea of Galilee).

##### Phenology.

Spring breeder, adults hibernate (pers. obs.).

##### Distribution range.

The species is distributed from Southwest Europe to Central Asia, southwards to Israel and Iran (Schmidt 2017). The subspecies syriacum is known from Southeast Turkey to Israel (Schmidt and Liebherr 2009) and from Iran (Schmidt 2017).

##### Distribution in the southern Levant.

In the southern Levant recorded exclusively from four sites in Israel, three of them from winter pools in the Central Coastal Plain (Berekhat Ya’ar (sometimes also written Breikhat Ya’ar), Dora Pool and Ga’ash Pool; from the latter two sites only single specimens). A large population exists also in the swamps north of the Sea of Galilee.

##### Taxonomic notes.

Schmidt (2017) listed the nominate subspecies from Israel, but we know only the subspecies subspecies syriacum from the southern Levant. All specimens we know from the southern Levant show a greenish metallic luster on the upper side, despite the indication in the original description.

##### Conservation.

In contrast to *A.nigrum* a rarely recorded species; we know only two larger populations (Sea of Galilee, Berekhat Ya’ar). Threatened by habitat loss, drainage, and water table drawdown.

#### Agonum (s. str.) nigrum

Taxon classificationAnimaliaColeopteraCarabidae

Dejean, 1828

E638D427-0B3F-5EC8-BAF0-C9E94C5FB964

##### Dispersal power.

Fully winged and flight-active (Chikatunov et al. 2006; pers. obs.).

##### Habitat.

A hygrophilous species in a wide range of habitats: from freshwater to brackish water habitats like winter ponds, shaded and unshaded streams, lakes, swamps, etc. Very abundant in the swamps north of the Sea of Galilee (Figs 31, 33).

##### Phenology.

Spring breeder (reproduction. March to May), adults hibernate.

##### Distribution range.

From western Europe to Central Asia, northwards to Central Europe, southwards to North Africa, eastwards to Central Asia (Schmidt 2017).

##### Distribution in the southern Levant.

From the Mediterranean climate zone to desert zone (Central Negev and Wadi Hasa). Egypt (Sinai, several sites: Schatzmayr 1936), Israel (Schmidt and Liebherr 2009), Jordan (Nasir and Katbeh-Bader 2017), Lebanon, Syria (Schmidt and Liebherr 2009).

We did not find the species in the extremely arid and hot areas around the Dead Sea or at the springs in the Arava Valley. Also in the light catches from the En Gedi Nature Reserve (kept in the ZSM), which cover the whole year, the species is missing like all other Platynini.

##### Conservation.

In contrast to some other *Agonum* species not threatened and still widespread. Found even in strongly urbanized areas, like a winter pond circled by highway loops north of Tel Aviv (pers. obs.).

#### Agonum (s. str.) rugicolle

Taxon classificationAnimaliaColeopteraCarabidae

Chaudoir, 1846

B9FE3727-2886-5013-95E5-476CA4B2FF30

##### Dispersal power.

Fully winged.

##### Habitat.

Winter ponds and small associated streams. The water reservoir of Merom Golan hosts also a population (Fig. 31).

##### Phenology.

Spring breeder.

##### Distribution range.

From Southeast Europe to Iran, not in North Africa (Schmidt 1992, 2017).

##### Distribution in the southern Levant.

North Israel (Upper Galilee, Hermon, Golan Heights).

##### Taxonomic notes.

Schmidt (1992) shows the extraordinary variability of the species in terms of both morphology and coloration.

#### Agonum (s. str .) sordidum

Taxon classificationAnimaliaColeopteraCarabidae

Dejean, 1828

31F9B782-8F80-5A14-9B15-C87560BDCE72

##### Dispersal power.

Fully winged and flight active.

##### Habitat.

Winter ponds and small associated streams. The water reservoir of Merom Golan hosts also a population (Fig. 31).

##### Phenology.

Spring breeder, adults hibernate.

##### Distribution range.

From Southeast Europe to Asia Minor and southern Levant (Schmidt 2017).

##### Distribution in the southern Levant.

Known only from Golan Heights.

#### Agonum (Olisares) viridicupreum

Taxon classificationAnimaliaColeopteraCarabidae

(Goeze, 1777)

54629504-92EF-59A8-999D-88635D5D872A

##### Dispersal power.

Fully winged and flight active (pers. obs.). A species with an abrupt northward distributional extension in Central Europe (Drees et al. 2011).

##### Habitat.

Wetlands, mostly winter ponds, rarely on riverbanks of streams or in semi-open floodplain woodlands (e.g., swamps with *Tamarix* and *Eucalyptus* north of the Sea of Galilee) (Figs 31, 33).

##### Phenology.

Spring breeder, tenerals at least sometimes also in March; adults hibernate.

##### Distribution range.

The nominate subspecies from Southwest Europe to West Siberia and southern Levant (including Sinai, Drees et al. 2011); in North Africa (without Egypt) and Central Asia two further subspecies (Schmidt 2017).

##### Distribution in the southern Levant.

In the Mediterranean climate zone (Lebanon, North Israel, Egypt: North Sinai) (Drees et al. 2011), not yet known from Jordan. The southern Levant is the rear edge of the distribution range of the nominate subspecies. In contrast to the leading edge no poleward shift has been observed in the southern Levant (Drees et al. 2011).

#### Anchomenus alcedo

Taxon classificationAnimaliaColeopteraCarabidae

Schmidt, 2014

7FAFBBD9-F54C-5150-A28B-8BD3B01BC9CA

##### Dispersal power.

Fully winged and flight active.

##### Habitat.

Riparian species of permanent and temporary streams, both sun-exposed and shaded, e.g., by oleander (*Neriumoleander*) and willows (*Salix* spp.).

##### Phenology.

At type locality a spring breeder with copulation in March and April, in May already tenerals; adults hibernate (pers. obs.).

##### Distribution range.

Endemic in a small range of the southern Levant (Schmidt 2014).

##### Distribution in the southern Levant.

Few localities from North Lebanon to the West Golan Heights (Schmidt 2014). The species lives in Israel not only in the type locality, but also on the main stream downwards of Banyas Waterfalls.

##### Conservation.

Due to excessive water withdrawal, streams in Israel and Lebanon are drying out faster, and their flood dynamics are changing. This results in a deterioration of the gravel banks, which largely become overgrown and are then no longer available as habitat for the species. Headwater areas are developed into recreational areas and naturally occurring microhabitats are destroyed (e.g., Banyas Waterfalls). Threatened due to the small range and poor conservation status of flowing waters in the southern Levant.

#### Anchomenus bellus

Taxon classificationAnimaliaColeopteraCarabidae

Schmidt, 2014

A5A63807-6ED9-50AC-97D5-8BDD3D6E43BD

##### Dispersal power.

Fully winged.

##### Habitat.

Unknown.

##### Phenology.

Unknown; types are from May and June (Schmidt 2014).

##### Distribution range.

Endemic in South and central East Turkey, probably also in Northwest Syria (Schmidt 2014, 2017).

##### Distribution in the southern Levant.

Unknown (Schmidt 2014).

#### Anchomenus dorsalis infuscatus

Taxon classificationAnimaliaColeopteraCarabidae

Chevrolat, 1854

61B8D22B-A579-5F37-AFB9-7DC122687FF2

##### Dispersal power.

Fully winged, apical wing part of variable size (Liebherr 1991), classified as macropterous by Desender (1989); flight-active (Desender 1989; Turin 2000).

##### Habitat.

In Europe a widespread species of open habitats, esp. of grassland (meadows, pastures) and arable fields (Lindroth 1985, 1986; Trautner and Rietze 2017). Probably an important antagonist of some pest species, especially of some aphids and weevils (Griffiths et al. 1985; Zaller et al. 2008). In the Levant, the species is mostly restricted to wetlands and esp. close to winter ponds, it seems not to occur in dry batha (Timm et al. 2009) (Figs 31, 33).

##### Phenology.

Spring breeder; adults hibernate (Larsson 1939; Lindroth 1945; Keckwitz 1980; Basedow 1994), also in the southern Levant (pers. obs.).

##### Distribution range.

From West Europe to Central Asia, southwards to North Africa, northwards to Scandinavia. The nominate subspecies in Turkey, Syria, and Cyprus (Austin et al. 2008; Schmidt 2017). The subspecies infuscatus exclusively in Israel, Jordan, Lebanon (Schmidt 2014, 2017), and on Sinai (Egypt; see below and Suppl. material 1).

##### Distribution in the southern Levant.

Mainly in the Mediterranean climate zone of the southern Levant, but a few specimens were collected in the Dead Sea Area (Nahal Qumeran, ‘Enot Zuqim, 1993) and in the southern mountains of Sinai (Jebel Katarina, Wadi Feiran, Wadi Wattir, 1971–1976).

#### Atranus ruficollis

Taxon classificationAnimaliaColeopteraCarabidae

(Gautier des Cottes, 1858)

6F50A950-16F0-5C41-B45D-0CEC83F4A468

##### Dispersal power.

Hind wings are longer than the elytra; we know of a flight observation from a car net (Michael Schuelke, personal observation). Boiteau et al. (2000) caught the similar American species, *A.pubescens* (Dejean, 1828), in flight interception traps.

##### Habitat.

Softwood floodplain woodlands with oleander (*Neriumoleander*), Oriental plane (*Platanusorientalis*), and willows (*Salix* spp.); mostly close to springs/sources and streams; often under deposited driftwood, in the litter layer, and on small shaded gravel banks. The same habitat is populated in Europe (Paill and Gunczy 2016) and Cyprus (pers. obs.) (Fig. 34).

##### Phenology.

All our specimens from Israel are from March to May; probably a spring breeder with summer larvae.

##### Distribution range.

From southwest Europe (Serrano 2013) to southwest Asia (Austin et al. 2008; Azadbakhsh and Nozari 2015; Schmidt 2017), northwards to southern Central Europe (Paill and Gunczy 2016), southwards to Sicily (Brandmayr et al. 2005), and probably North Africa (Algeria, Lindroth 1968: 649).

##### Distribution in the southern Levant.

First record from Tanur (Tanur Waterfalls, Nahal ‘Iyyon Nature Reserve) (Wrase 2009), also in the wadis Nahal Kziv and Nahal Betzet (see Suppl. material 1). Probably also in Lebanon, but no records yet.

##### Conservation.

The damages to water bodies of streams (exploitation, pollution, reduced dynamics of floods due to drainage) (Goren 2004) has probably resulted in a decrease of habitats for *A.ruficollis*. In recent years (2018, 2019), chlorinated water has also been used to feed the stream in the Nahal Betzet (pers. obs.).

#### Olisthopus fuscatus

Taxon classificationAnimaliaColeopteraCarabidae

Dejean, 1828

EA199DEE-5E99-53B4-BDE8-EBA67AAC7EB8

##### Dispersal power.

Fully winged (n = 4) and flight activity (at light, own observation from Spain).

##### Habitat.

In open, sparsely vegetated habitats on loamy or sandy soil, also in clearings of forests. Apparently not restricted to wetlands, even if the species is found there from time to time. (Campos Gómez and Novoa Docet 2006; Teofilova 2018; Jiroux 2019; pers. obs. from southern Europe and southern Levant).

##### Phenology.

Unknown.

##### Distribution range.

From southwest Europe (Spain) to Asia minor and southern Levant, including Cyprus, southwards to North Africa (Austin et al. 2008; Serrano 2013; Schmidt 2017).

##### Distribution in the southern Levant.

In the Mediterranean part of Israel (Upper Galilee, Lower Galilee, Golan Heights, Carmel Ridge, Central coastal Plain, Judean Hills). Already mentioned by Bodenheimer (1937) and Nitzu (1995) from Israel. New records for Lebanon (16 km S Tripoli, Amioun, 7.v.2011, K. Orszulik leg., 1 male, CWGP), and Jordan (ca 20 km N Amman, 250 m, 32°12.906'N, 35°53.093'E, 19.v.2007, Zb. Kejval leg., 1 female, CWGP).

##### Taxonomic notes.

The tip of the median lobe of the aedeagus shows remarkable variation across the distribution range, but the endophallus seems to be of the same shape. Coulon et al (2011) offer figures of the median lobe of the aedeagus of both *O.fuscatus* and *O.glabricollis*.

#### Olisthopus glabricollis

Taxon classificationAnimaliaColeopteraCarabidae

(Germar, 1817)

ABA48890-CFB2-574D-9664-A8BCD0981C87

##### Dispersal power.

Fully winged (n = 8) and probably flight-active.

##### Habitat.

Batha (with *Sarcopotheriumspinosum*) and oak woodlands (with *Quercuscalliprinos*, *Q.boissieri*), also grazed sites (Kaltsas et al. 2013; own observation in southern Levant); in Bulgaria in grasslands (Teofilova 2018). Although the species can occur close to puddles and temporary ponds (e.g., in the Ya’ar Odem), it is not restricted to wetlands as it is known from most *Agonum* species.

##### Phenology.

Probably an autumn and winter breeder. Timm et al. (2009) caught three individuals in a survey with pitfall traps open continuously for an entire year and these exclusively in November and December. A newly hatched beetle at the end of March on the Golan Heights. Thirty-six specimens collected on the southern slopes of the Judean Hills in 2001–2004 were collected between November and April, predominantly in November (SMNHTAU).

##### Distribution range.

From southwestern Europe to Asia Minor and southern Levant (Bodenheimer 1937; Schmidt 2017). New record for Iraq: N Iraq, Guly Ali Beq, 1400 m,1.v. 2006, C. Reuter leg., 1 male, CWGP.

##### Distribution in the southern Levant.

Few records from Israel (Bodenheimer 1937; Timm et al. 2008). Recently found in Upper Galilee, Golan Heights, Carmel Ridge, Central Coastal Plain, and Judean Hills (Suppl. material 1).

#### Orthotrichus cymindoides

Taxon classificationAnimaliaColeopteraCarabidae

(Dejean, 1831)

2B16E191-EA98-5429-808C-A70B75CD74D4

##### Dispersal power.

Fully winged (n = 4).

##### Habitat.

In Pakistan, in thorn scrub forests (Ullah 2017).

##### Phenology.

In Saudi Arabia, from November to May (Abdel-Dayem et al. 2017).

##### Distribution range.

From Egypt to Iran and Pakistan (Schmidt 2017; Ullah 2017).

##### Distribution in the southern Levant.

Known from Egypt, incl. Sinai (Schatzmayr 1936; Alfieri 1976; Abdel-Dayem 2004) and Syria (Schmidt 2017).

## Discussion

### The identification tools

Computer- and/or Internet-based identification options are increasing. Many of them work very well including, for example, some applications for smartphones, tablets, or personal computers. Some of these applications are based on machine learning (esp. convolutional neural network, CNN) (cf. https://plantnet.org/en/). However, the results from CNN published to date for ground beetles seem not to be suitable for taxonomic identifications. For example, with the approach taken by Hansen et al. (2019), many species can only be identified with high error probability. It will probably be some time before CNN-based identification tools for ground beetles with their many sibling species are available and provide good identification results. Meanwhile, interactive identification keys may be helpful as an alternative to classical dichotomous identification keys.

The keys presented here have different numbers of decisions up to species identification. Especially the interactive key allows a fast identification. However, one character, the coloration of the upper side, has many states. In case of polychromatic species, the coloration can only be used to unambiguously identify some individuals of a species. This applies for example to the very variable species *Agonumrugicolle*. The black color morph of the latter species is, regarding the coloration, similar to the “dark” *Agonum* species like *A.nigrum* and *A.monachumsyriacum*. For species where some color variants occur very rarely (e.g., black variants in *Agonummarginatum*; Schmidt 1992), these were not considered in the key. However, the additional morphological criteria allow an identification in further steps.

We do not know whether the identification of ground beetle species with the help of an interactive key is as reliable as with a classical dichotomous key. Up to now, we had the impression that an identification is more reliable if a combination of character states leads to the actual result as typically encounter in classical dichotomous identification key. Often, features such as coloration or body length can validate an identification. If one would like to have this additional information with the use of the interactive key, one should consult additional tools of our Xper3 knowledge base. These include the information that can be found in the species (“items”) section under “Details” and photos under “Pictures”. In addition, under the tools there is also the “Description Matrix” with the character states for each species and character.

Interactive and single access keys, created by Xper3, seem to have a greater efficiency than classical dichotomous keys, not only in our study, but also for coccinellids in France (Jouveau et al. 2018). However, it may be possible that the use of dichotomous identification keys, which group species by their higher taxonomic units (species groups, subgenera, genera, etc.), may also have an educational impact on the users. Thus, in addition to identifying species, dichotomous keys could also provide a better understanding of the relationships of taxa. To our knowledge, there have been no studies of such potential effects. The same is true for establishing taxonomic knowledge through different types of keys. Given the dramatic decline of taxonomic knowledge in large parts of the public, including its academic parts (Kim and Byrne 2006; Coleman and Radulovici 2020), such studies seem to be important.

We see a great advantage of knowledge bases created on the Xper3 platform in the possibility to remain dynamic. This implies that a knowledge base could be updated when new knowledge is available, e.g., by adding further taxa. In the case of the knowledge base ‘Platynini, southern Levant’, it can start with adding the diagnostic features of the Platynini (as listed in this paper) for each species and subspecies. Afterwards, further taxa of other tribes can be added. Since several experts can work simultaneously on the database and the generating of the identification tools works, the structured Xper3 knowledge database seems to us very suitable for the taxonomic treatment of species-rich faunas in particular in areas where knowledge is limited and can change rapidly such as in the Middle East.

### Distribution, habitat preference, and reproduction rhythms

At least eight species of Platynini, and thus their majority in the Levant, are associated with high ground-water tables or running water bodies as part of their habitats. These species also appear to be (predominantly) spring breeders. This is exceptional for ground beetles in such summer-dry areas with Mediterranean or desert climates. Most ground beetles found there reproduce in the fall and winter and larval development occurs during the winter months when precipitation is high (Paarmann 1970, 1979; Brandmayr et al. 1983; Liebherr 1984; Pizzolotto et al. 2018). However, the high water availability in their habitats allows ground beetles in such habitats to develop in spring and early summer. This is also consistent with the reproductive season of other hygrophilous ground beetle species in the southern Levant (Assmann et al. 2015) and in the Cyrenaica (Paarmann 1970). Under similar climatic conditions in North America, e.g., in California, some *Agonum* species occur and they all hibernate as imagines, reproduce in spring, and develop as larvae in summer (Liebherr 1983; Larochelle and Larivière 2003).

The reproductive period of the two *Olisthopus* species in the Levant is not yet sufficiently clear, but first indications suggest reproduction during autumn and winter. From *Olisthopusrotundatus* in Central Europe and Scandinavia it is known that adults reproduce in autumn and larval development takes place during winter and spring; tenerals occur in summer (Lindroth 1985 and 1986; Homburg et al. 2014). At least some of the North American *Olisthopus* species reproduce during spring (Larochelle and Larivière 2003; Lindroth 1966). Both *Olisthopus* species in the Levant seem to be not so strongly restricted to wet habitats as it is known from *Atranusruficollis* and the *Agonum* species (see species accounts).

A complex system with different photoperiodic control of previtellogenesis and vitellogenesis (and probably also spermatogenesis) determines the reproductive rhythmicity of many ground beetles (Thiele 1977). A genetic basis for this eco-physiological phenomenon is thus very likely. In general, closely related species share more similar species traits than less related species (Harvey and Pagel 1991;Webb et al. 2002). Species traits like reproduction rhythms can be based by phylogenetic effects (Purvis 2008). The coincidence in reproductive rhythmicity among *Agonum* species in the Levant, but also far beyond (e.g., Lindroth 1985,1986; Larochelle and Larivière 2003; Homburg et al 2014; Trautner and Rietze 2017), can therefore be interpreted as a trait evolved by a common ancestor. This is supported by the fact that the genus *Agonum* undoubtedly represents a monophylum that includes the subgeneric classification adopted here (Liebherr and Schmidt 2004, 2005). For ground beetles it should be tested if phylogeny has an impact on the formation of the traits. To our knowledge, such a test is still missing. If we assume that phylogeny has an effect on traits, then phylogeny should also have an indirect effect on species composition and population dynamic processes that are influenced by traits. However, no or very small effects of species’ phylogenetic relatedness was found for the habitat preferences of ground beetles or community composition (Davies et al. 2000; Kotze and O’Hara 2003; Nolte et al 2017, 2019).

### Conservation, habitat loss, and indicator value

Many *Agonum* species depend on winter ponds. These are seasonal ponds, which are filled during winter. In summer, the ponds dry up and lay “dormant”. These habitats are among the most threatened habitats in the Levant. In the Coastal Plain of Israel, more than 80% of these semi-aquatic habitats have disappeared during the last decades (Levin et al. 2009). Moreover, the remnants of these habitats are more isolated today than they were a few decades ago. We suspect a higher probability of progressive species declines and loss at the remaining habitat remnants. For some animal groups such a decline could already be proven (Doley and Perevolotsky 2004). Endangered ground beetles also inhabit vernal pools in California, which has a climate similar to parts of the southern Levant and has comparable land use changes. These species include the well-known ground beetle *Elaphrusviridis*, which is on the U.S. Endangered Species List (Goulet 1983; Larochelle and Larivière 2003; Erwin 2011).

Continued water withdrawal and groundwater lowering are severely threatening the stream habitats in the Levant (Goren 2004). This also threatens *Anchomenusalcedo*, which is endemic to Lebanon and Israel. Both countries have a national responsibility for the global conservation of this species (cf. Schnittler and Günther 1999; Schuldt and Assmann 2010; Schmidt and Trautner 2016). The same is true for other ground beetle species that have a small range in the southern Levant and are restricted to highly threatened habitats (Assmann et al. 2018; Renan et al. 2018).

The hygrophilous *Agonum* and *Anchomenus* species provide an indication potential for the extent of wetland disturbance or loss of such habitats (cf. Bonn et al. 2002; Koivula 2011). Also in restoration ecology, these ground beetles have an outstanding indication potential (Günther and Assmann 2005; Gerisch et al. 2006; Januschke et al. 2011). With our identification tools, ecological and distributional traits, but also the database of records, we hope to establish a baseline for further studies, esp. long-term monitoring of these ground beetles in the southern Levant.

## Supplementary Material

XML Treatment for Agonum (s. str.) marginatum

XML Treatment for Agonum (s. str.) mesostictum

XML Treatment for Agonum (s. str.) monachum syriacum

XML Treatment for Agonum (s. str.) nigrum

XML Treatment for Agonum (s. str.) rugicolle

XML Treatment for Agonum (s. str .) sordidum

XML Treatment for Agonum (Olisares) viridicupreum

XML Treatment for Anchomenus alcedo

XML Treatment for Anchomenus bellus

XML Treatment for Anchomenus dorsalis infuscatus

XML Treatment for Atranus ruficollis

XML Treatment for Olisthopus fuscatus

XML Treatment for Olisthopus glabricollis

XML Treatment for Orthotrichus cymindoides

## Supplementary materials

Supplementary material 1 Material examined: Records of the Platynini from the southern Levant hosted in studied collections (see for abbreviation: Materials and methods) Data type table https://binary.pensoft.net/file/554583This dataset is made available under the Open Database License (http://opendatacommons.org/licenses/odbl/1.0/). The Open Database License (ODbL) is a license agreement intended to allow users to freely share, modify, and use this Dataset while maintaining this same freedom for others, provided that the original source and author(s) are credited.Thorsten Assmann, Estève Boutaud, Jörn Buse, Ariel-Leib-Leonid Friedman, Ingmar Harry, Fares Khoury, Eylon Orbach, Ittai Renan, David W. Wrase, Pascale Zumstein

Supplementary material 2 Single access identification key generated by Xper3, using weights of the characters and prioritization of characters with few states Data type Hypertext Markup Language (html) https://binary.pensoft.net/file/554584This dataset is made available under the Open Database License (http://opendatacommons.org/licenses/odbl/1.0/). The Open Database License (ODbL) is a license agreement intended to allow users to freely share, modify, and use this Dataset while maintaining this same freedom for others, provided that the original source and author(s) are credited.Thorsten Assmann, Estève Boutaud, Jörn Buse, Ariel-Leib-Leonid Friedman, Ingmar Harry, Fares Khoury, Eylon Orbach, Ittai Renan, David W. Wrase, Pascale Zumstein

Supplementary material 3 Exported knowledge database ‘Platynini, southern Levant’ file in sdd generated by Xper3 Data type Extensible Markup Language (xml) https://binary.pensoft.net/file/557946This dataset is made available under the Open Database License (http://opendatacommons.org/licenses/odbl/1.0/). The Open Database License (ODbL) is a license agreement intended to allow users to freely share, modify, and use this Dataset while maintaining this same freedom for others, provided that the original source and author(s) are credited.Thorsten Assmann, Estève Boutaud, Jörn Buse, Ariel-Leib-Leonid Friedman, Ingmar Harry, Fares Khoury, Eylon Orbach, Ittai Renan, David W. Wrase, Pascale Zumstein
