# Supplementary figures and images for: The ground beetle tribe Platynini Bonelli, 1810 (Coleoptera, Carabidae) in the southern Levant: dichotomous and interactive identification tools, ecological traits, and distribution
Source: Zookeys. 2021 Jun 16;1044:449–78. doi: 10.3897/zookeys.1044.62615 (PMC9425446; doi:10.3897/zookeys.1044.62615)

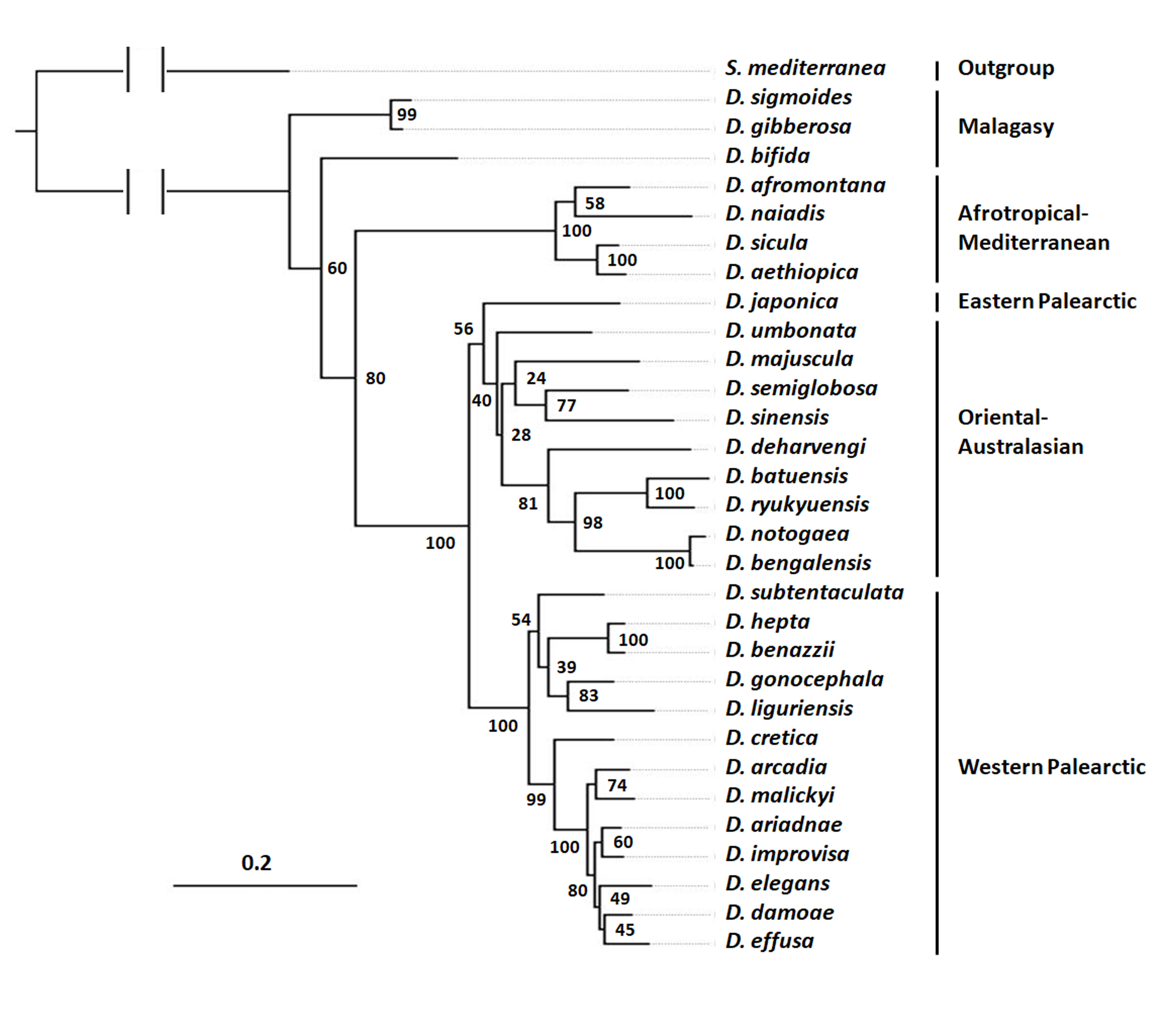

Supplement: Supplementary material 1 — Material examined: Records of the Platynini from the southern Levant hosted in studied collections (see for abbreviation: Materials and methods) [file zookeys-1044-449-s001.tif]
