# Supplementary material for: The ground beetle tribe Platynini Bonelli, 1810 (Coleoptera, Carabidae) in the southern Levant: dichotomous and interactive identification tools, ecological traits, and distribution
Source: Zookeys. 2021 Jun 16;1044:449–78. doi: 10.3897/zookeys.1044.62615 (PMC9425446; doi:10.3897/zookeys.1044.62615)
Supplement: Supplementary material 2 — Single access identification key generated by Xper3, using weights of the characters and prioritization of characters with few states [file zookeys-1044-449-s002.html]

Platynini, southern Levant, Generated by IKey+, Laboratoire Informatique et Systematique, UMR 7205, MNHN Paris  
  
Options:  
sddURL=https://www.xper3.fr:443/xper3GeneratedFiles/sdd/export/Platynini\_\_southern\_Levant-7F07FB9CBDB8D9F18E5B846838806E75.sdd  
format=html  
representation=flat  
fewStatesCharacterFirst=true  
mergeCharacterStatesIfSameDiscrimination=true  
pruning=true  
verbosity=ho  
scoreMethod=xper  
weightContext=ObservationConvenience  
weightType=global  
  
parseDuration= 0.195s  
keyCreationDuration= 0.029s  
  
  
**1** Pronotum: hind angles :  
                  rounded, hind angles not prominent => 2  
                  sharp or obtuse, but hind angles clearly recognizable => 3  
  
**2** Body length :  
                  [5.0, 8.5[ (mm) => 4  
                  [8.5, 12.0] (mm) => 5  
  
**3** Hairs on upper side (pronotum, elytra) :  
                  absent (only the regular setae, supraorbital setae, series umbilicata etc.) => 6  
                  present => Atranus ruficollis (Gautier des Cottes, 1858)  
  
**4** Body length :  
                  [5.0, 7.0[ (mm) => 7  
                  [7.0, 10.0] (mm) => 8  
  
**5** Hairs on upper side (pronotum, elytra) :  
                  absent (only the regular setae, supraorbital setae, series umbilicata etc.) => 9  
                  present => Orthotrichus cymindoides (Dejean, 1831)  
  
**6** First antennomere :  
                  in apical part enlarged, behind a small constriction => Anchomenus alcedo Schmidt, 2014  
                  regularily formed, in apical part neither with a constriction nor widened => 10  
  
**7** Mentum (**?**):  
                  with tooth => Agonum (s.str.) mesostictum (Bates, 1889)https://zenodo.org/api/iiif/v2/ffb0515c-69e2-47a9-a080-4db3d07a367a:b31339cc-1611-4ffc-9bc6-c16cbe128223:Agonum\_rugicolle\_mentum.png/full/750,/0/default.png  
                  without tooth => 11https://zenodo.org/api/iiif/v2/34249830-c543-4931-ab71-7f0f73d08471:54276f76-9eac-4ff7-a276-f11e9fa75b5c:Mentum\_Olisthopus\_glabricollis.png/full/750,/0/default.png  
  
**8** Pronotum: lateral margin :  
                  lateral sides convex, but straight (and converging) in the basal half => 12  
                  lateral sides continuously convex => 13  
  
**9** Pronotum: lateral margin :  
                  lateral sides convex, but straight (and converging) in the basal half => 14  
                  lateral sides continuously convex => 15  
  
**10** Coloration of upper side :  
                  forebody green to bluish green, elytra reddish to brown with darkening on disc => Anchomenus dorsalis infuscatus Chevrolat, 1854  
                  blue to violett => Anchomenus bellus Schmidt, 2014  
  
**11** Pronotum: punctation :  
                  strong punctation => Olisthopus fuscatus Dejean, 1828  
                  weak punctation => Olisthopus glabricollis (Germar, 1817)  
  
**12** Metallic luster of upper side :  
                  strong => Agonum (Olisares) viridicupreum (Goeze, 1777)  
                  weak (fair metallic sheen) => Agonum (s.str.) mesostictum (Bates, 1889)  
                  absent => Agonum (s.str.) mesostictum (Bates, 1889)  
  
**13** Coloration of legs :  
                  dark brown or black => Agonum (s.str.) nigrum Dejean, 1828  
                  dark brown, femur darker than tibia => Agonum (s.str.) nigrum Dejean, 1828  
                  femur reddish to brownish, rarely dark-brown, tibia and tarsus darker => Agonum (s.str.) sordidum Dejean, 1828  
                  yellow, reddish or brownish (brightened) => Agonum (s.str.) sordidum Dejean, 1828  
  
**14** 5th elytral stria and adjacent intervals :  
                  depressed => 16  
                  not depressed => Agonum (Olisares) viridicupreum (Goeze, 1777)  
  
**15** Coloration of legs :  
                  dark brown or black => Agonum (s.str.) nigrum Dejean, 1828  
                  dark brown, femur darker than tibia => Agonum (s.str.) nigrum Dejean, 1828  
                  femur reddish to brownish, rarely dark-brown, tibia and tarsus darker => Agonum (s.str.) sordidum Dejean, 1828  
                  yellow, reddish or brownish (brightened) => 17  
  
**16** Pronotum: proportion :  
                  pronotum very wide, > 1.3 times wider than long => Agonum (s.str.) rugicolle Chaudoir, 1846  
                  pronotum very slender, < 1.12 wider than long => Agonum (s.str.) monachum syriacum Schmidt, 2009  
  
**17** 5th elytral stria and adjacent intervals :  
                  depressed => Agonum (s.str.) sordidum Dejean, 1828  
                  not depressed => Agonum (s.str.) marginatum (Linnaeus, 1758)
